# 

**Published:** 1898-07

**Authors:** 


ESTABLISHED 1855. SUBSCRIPTION, $2.00.

### ATLANTA MEDICAL AND SURGICAL JOURNAL.

SURGEON GENERAL'S OFFICE
JUL.-14-1898

VOLUME XV NEW SERIES. Atlanta, Ga., July, 1898. No. 5.

